# CaMKIIα may modulate fentanyl-induced hyperalgesia via a CeLC-PAG-RVM-spinal cord descending facilitative pain pathway in rats

**DOI:** 10.1371/journal.pone.0177412

**Published:** 2017-05-10

**Authors:** Zhen Li, Pingping Yin, Jian Chen, Shenglan Jin, Jieqiong Liu, Fang Luo

**Affiliations:** 1 Department of Anesthesiology, Tongji Hospital, Tongji Medical College, Huazhong University of Science and Technology, Wuhan, China; 2 The Laboratory of Membrane Ion Channels and Medicine, Key Laboratory of Cognitive Science, State Ethnic Affairs Commission, College of Biomedical Engineering, South-Central University for Nationalities, Wuhan, China; 3 Department of Anesthesiology, The Central Hospital of Wuhan, Tongji Medical College, Huazhong University of Science and Technology, Wuhan, China; University of Texas Medical Branch at Galveston, UNITED STATES

## Abstract

Each of the lateral capsular division of central nucleus of amygdala(CeLC), periaqueductal gray (PAG), rostral ventromedial medulla(RVM) and spinal cord has been proved to contribute to the development of opioid-induced hyperalgesia(OIH). Especially, Ca^2+^/calmodulin-dependent protein kinase II*α* (CaMKIIα) in CeLC and spinal cord seems to play a key role in OIH modulation. However, the pain pathway through which CaMKIIα modulates OIH is not clear. The pathway from CeLC to spinal cord for this modulation was explored in the present study. Mechanical and thermal hyperalgesia were tested by von Frey test or Hargreaves test, respectively. CaMKIIα activity (phospho-CaMKIIα, *p*-CaMKIIα) was evaluated by western blot analysis. CaMKIIα antagonist (KN93) was micro-infused into CeLC, spinal cord or PAG, respectively, to evaluate its effect on behavioral hyperalgesia and *p*-CaMKIIα expression in CeLC, PAG, RVM and spinal cord. Then the underlying synaptic mechanism was explored by recording miniature excitatory postsynaptic currents (mEPSCs) on PAG slices using whole-cell voltage-clamp methods. Results showed that inhibition of CeLC, PAG or spinal CaMKIIα activity respectively by KN93, reversed both mechanical and thermal hyperalgesia. Microinjection of KN93 into CeLC decreased *p*-CaMKIIα expression in CeLC, PAG, RVM and spinal cord; while intrathecal KN93 can only block spinal but not CeLC CaMKIIα activity. KN93 injected into PAG just decreased *p*-CaMKIIα expression in PAG, RVM and spinal cord, but not in the CeLC. Similarly, whole-cell voltage-clamp recording found the frequency and amplitude of mEPSCs in PAG cells were decreased by KN93 added in PAG slice or micro-infused into CeLC in vivo. These results together with previous findings suggest that CaMKIIα may modulate OIH via a CeLC-PAG-RVM-spinal cord descending facilitative pain pathway.

## Introduction

Opioids can provide effective pain relief but their use is limited because of a clinical paradoxical syndrome in which patients on long-term opioids exposure become more sensitive to pain. This paradoxical phenomenon has been named as opioid-induced hyperalgesia (OIH) [1–3]. OIH has been reliably demonstrated not only in humans suffered from different types of pain but also in healthy volunteers and opioid addicts [4–6]. Several rodent models have also been setup to facilitate the study of this phenomenon. Morphine and remifentanil induced hyperalgesia have been largely explored at spinal level as well as superspinal level. Recently, we have explored the mechanism of fentanyl-induced hyperalgesia at both spinal cord and the lateral capsular division of central nucleus of amygdala(CeLC). In addition, we have found a pivotal role of Ca^2+^/calmodulin-dependent protein kinase II*α* (CaMKIIα) in the modulation of OIH.

CaMKIIα, contributing to hyperalgesia priming[7], is a multifunctional serine/threonine protein kinase that has an outstanding effect on glutamate neurotransmission and behavior-related synaptic plasticity [8, 9], which makes it a very important target to explore the neural mechanisms of OIH. Although the underlying mechanism of OIH is not yet completely understood[1, 3, 4, 10], previous reports from our groups have shown that CaMKIIα, which is highly expressed in the CeLC and superficial spinal dorsal horn, plays an important role in modulation of OIH[11, 12]. Either spinal or CeLC CaMKIIα inhibition can attenuate OIH [11, 12]. The descending pain modulation from the periaqueductal gray (PAG) and rostral ventromedial medulla(RVM) has clearly been involved in the development of hyperalgesia and analgesic tolerance induced by opiates [13–16]. Local administration of lidocaine into RVM abolished OIH in rat[17]. In the PAG also, Toll Like Recepter-4 signaling, phosphorylation of mitogen-activated protein kinase and c-Fos have been shown to participate in OIH [18–21]. CeLC, a target of the spino-parabrachio-amygdaloid pain pathway that has been considered as “the nociceptive amygdala” [22–24], is also the upstream of PAG-RVM-spinal cord descending pain pathway[22, 25–29]. Anatomical studies have demonstrated direct reciprocal neurons projections between the amygdala and the PAG [30, 31]. Given the close anatomical connectivity between these regions and their role in OIH pain modulation, we speculate that there may exist a CeLC-PAG-RVM-spinal cord descending pathway to modulate OIH.

The purpose of this study was to explore whether CaMKIIα modulates OIH via a CeLC-PAG-RVM-spinal cord descending pathway. In our previously experiment, we have found that detectable mechanical and thermal behavioral hyperalgesia peaked at 6.5 h post fentanyl injection, and lasted for 3 d-4 d [11]. Therefore, 6.5 h post fentanyl injection, the CaMKIIα inhibitor KN93 was micro-infused into CeLC, spinal cord and PAG respectively to evaluate its effect on behavioral hyperalgesia and the activity of CaMKIIα at different level of the CeLC-PAG-RVM-spinal cord descending pathway. Finally, the underlying synaptic mechanism was explored by recording miniature excitatory postsynaptic currents(mEPSCs), which are frequently used as a parameter to reflect altered synaptic transmission [32, 33], on the PAG slices using whole-cell voltage-clamp methods.

## Materials and methods

### Ethics statement

All procedures were approved by and implemented in accordance with the Institutional Animal Care and Use Committee of Tongji Hospital, Tongji Medical College, Huazhong University of Science and Technology (Permit Number: TJ- A20141217), and performed in the light of the guides and policies by the International Association for the Study of Pain[34]. Rats were sacrificed by subsequent decapitation after sodium pentobarbital (50 mg/kg, i.p.) and all available efforts were for minimizing animal suffering.

### Animal care and utilization

Male Sprague-Dawley rats (80-120g, obtained from animal laboratory of Tongji Medical College, Huazhong University of Science and Technology, Wuhan, China) were maintained on a 12:12 h light-dark cycle in a climate-controlled room (24°C) with food and water available *ad libitum*. We monitored the physical condition of the experiment animals every day and none of the experiment animals became severely ill or died at any time. Intraperitoneal administration of sodium pentobarbital (50 mg/kg) was used to anaesthetize the experiment animals.

### Experimental protocol and drug delivery

**Fig 1:**
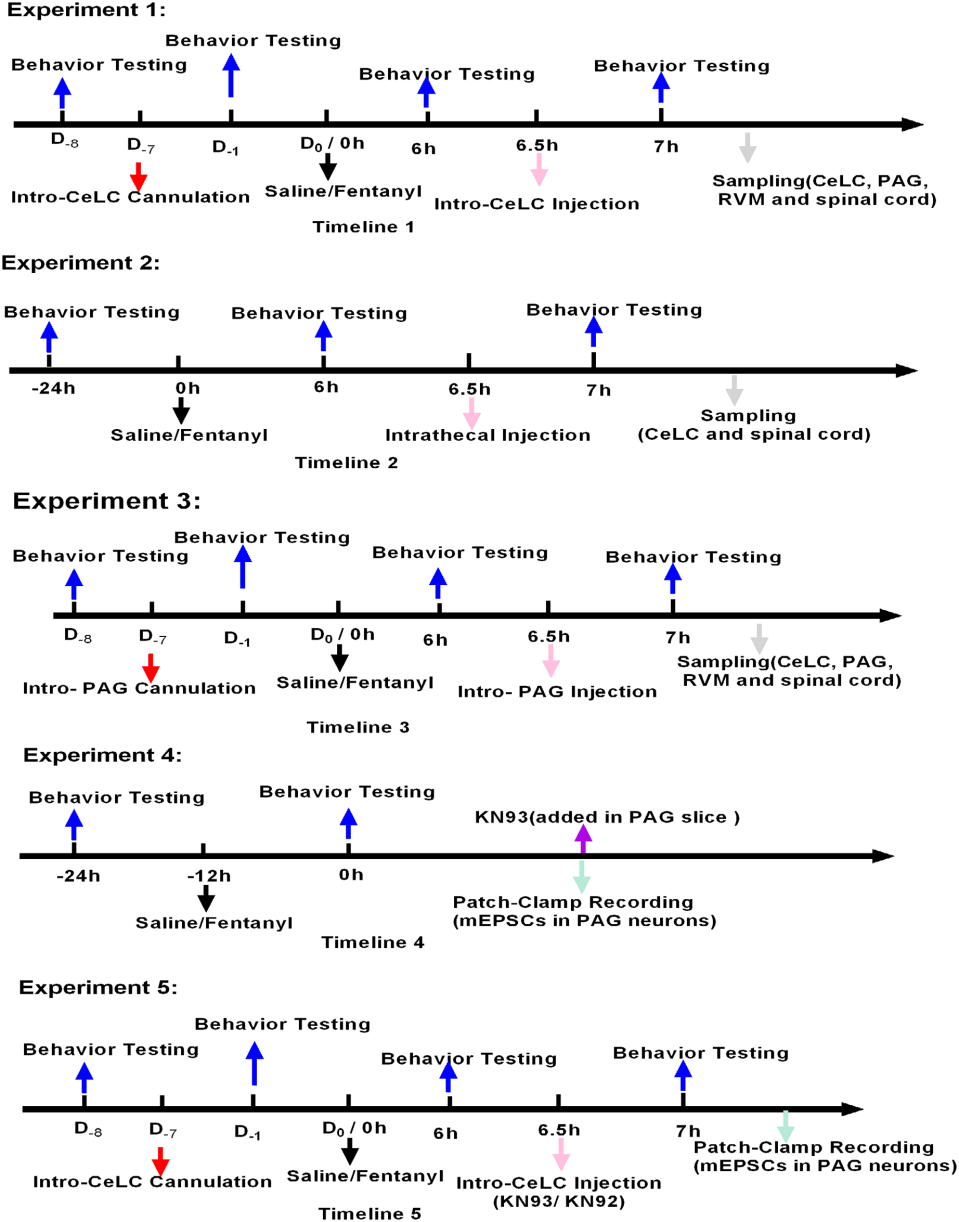
Illustration of the experimental design. Experiment 1: To investigate the effect of CeLC CaMKIIα antagonism on the level of CaMKIIα activity in CeLC, PAG, RVM and spinal cord regions and the behavioral hyperalgesia induced by fentanyl, rats were first implanted with CeLC cannulas before the induction of OIH by fentanyl. Then the Control group (n = 6) and OIH group (n = 6) received 50%DMSO (vehicle) 0.3 μl. OIH+KN92 10 nmol group (n = 6) and OIH+KN93 10 nmol group (n = 6) received equal volume (0.3 μl) of KN92 10 nmol and KN93 10 nmol respectively 6.5 h after the last injection of fentanyl. Experiment 2: To test whether spinal cord has a reverse effect on amygdala CaMKIIα signal, animals were randomly divided into four groups (n = 6). The Control group and OIH group received 50% DMSO (vehicle) 0.45 μl. OIH+KN92 45 nmol group and OIH+KN93 45 nmol group received equal volume (0.45 μl) of KN92 45 nmol and KN93 45 nmol respectively 6.5 h after the last injection of fentanyl. Experiment 3: To confirm whether *p-*CaMKIIα in vlPAG is involved in fentanyl-induced hyperalgesia and whether there is connection between PAG and other regions including CeLC, RVM and spinal cord in OIH, animals were randomly divided into four groups (n = 6). Control group and OIH group received 50% DMSO (vehicle) 0.3 μl. OIH + KN92 10 nmol group and OIH + KN93 10 nmol group received KN92 10 nmol and KN93 10 nmol respectively 6.5 h after the last injection of fentanyl. Experiment 4: To determine if there were differences between the Control rats and OIH rats in the synaptic transmission, and whether CaMKIIα modulates synaptic transmission in PAG neurons in OIH rats, the PAG slices of control rats (n = 6) and OIH rats (n = 6) were obtained 12 h after the last injection of saline or fentanyl. KN93 (10 μM) were added in the recording well 10 min after the baseline recording. Experiment 5: To further confirm whether inhibition of CeLC CaMKIIα activity has a direct effect on the enhanced synaptic transmission of vlPAG neurons in OIH rats, the Control+KN92 (n = 11) group and OIH+KN92 (n = 8) received CeLC injection of KN92 (10 nmol) and Control+KN93 group (n = 8) and OIH+KN93 (n = 8) group received CeLC injection of KN93 (10 nmol) respectively 6.5 h after the last injection of saline or fentanyl.

The detailed experimental design was illustrated in Fig 1. Chemicals other indicated were obtained from Beyotime and Boster (Shanghai, China). Four times injections of fentanyl (60 μg/kg per injection, s.c.) at 15 min intervals, resulting in a cumulative dose of 240 μg/kg [10, 11] were conducted to induce OIH in this study. The control animals received an equal volume (1.2 ml/kg) of physiological saline. KN93(N-[2-[[[3-(4’-Chlorophenyl)-2-propenyl]methylamino]methyl]phenyl]-N-(2-hydroxyethyl)-4′-methoxybenzenesulfonamide phosphate salt) and KN92 (2-[N-(4-methoxybenzenesulfonyl)]amino-N-(4-chlorocinnamyl)-N-methylbenzylamine, monohydrochloride)were purchased from Cayman (Ann Arbor, MI) and were dissolved in 50% dimethyl sulfoxide (DMSO). 50% DMSO was used as a vehicle control. Intrathecal (i.t.) injections were performed manually between the L5 and L6 inter-vertebral space in conscious rats as previously described [12]. The injection was performed using a glass microsyringe (RWD Life Science, Shenzhen, China). Each rat was injected with a volume of 0.45 μl. Success of the i.t. injection was verified by a lateral tail-flick.

### Behavior testing

#### Mechanical hyperalgesia

Mechanical sensitivity was tested using the von Frey filaments (North Coast, San Jose, CA, USA) according to the Dixon method[35, 36]. Before the test, rats were placed into separate Plexiglas containers over mesh platforms and allowed to adapt about 30 min to achieve immobility. Beginning with 1.0 g, fibers of sequentially increasing stiffness were perpendicularly applied to the mid-plantar surface according to the up-down paradigm. Positive response was defined as paw flinching shook, or licking its paw or brisk withdrawal. The interval between adjacent tests was more than 5 minutes. The 50% probability of paw withdrawal threshold was calculated using the up and down paradigm [11, 36, 37].

#### Thermal hyperalgesia

Rats were habituated to the test environment for 30 min before assessment. Thermal sensitivity was assayed with a radiant thermal stimulator (BME-410C, Biomedical Engineering, Boerni science and technology limited company, Guangzhou, China) according to the Hargreaves’ method [35, 36, 38]. The time appearing positive response, which was defined as a clear paw withdrawal, was recorded as the paw withdrawal latency after the application of radiant thermal stimulator to the mid-plantar surface of the left hind paw through the glass floor. The cutoff was 15 s to prevent damaging of tissues. The test was repeated three trials with a 5-minute interval, and then the thermal latency was defined as the mean of three trials [11].

### Western blotting

The proteins (see flowchart illustrated in Fig 1) from each group after the behavioral test were extracted using the radio-immuno-precipitation assay buffer and protein concentration was determined by bicinchoninic acid assay [11, 12]. Samples (20 μg of total protein) were separated by 10% sodium dodecyl sulfate polyacrylamide gel electrophoresis and transferred electrophoretically onto polyvinylidene fluoride membrane. After blocking with 5% skim milk for 2 h at room temperature, the membranes were incubated in primary rabbit anti-(T286)*p-*CaMKIIα antibody (1:1,000; Santa Cruz Biotechnology, Santa Cruz, CA) or a mouse anti-GAPDH antibody (1:400; Boster, Shanghai, China) at 4°C overnight. After having been washed, the membranes were incubated with HRP conjugated anti-rabbit (for *p-*CaMKIIα) or anti-mouse (for GAPDH) secondary antibody IgG (1:10,000, Boster, Shanghai, China) at room temperature for 90 min. The *p-*CaMKIIα antibody detected double bands in the experiments, both of which correspond to *p-*CaMKIIα[39]. The immunoblots were visualized by chemiluminescence or enhanced chemiluminescence signals (Thermo Fisher, Shanghai, China) detection using a Bio-Rad ChemiDoc (Shanghai, China) system and normalized to GAPDH bands.

### Cannulation and microinjection

Briefly, rats were deeply anesthetized with sodium pentobarbital (50 mg/kg, i.p.) and mounted in a stereotaxic frame (Zenda, Austin, Texas, USA. As reported, the guide cannula (RWD Life Science, Shenzhen, China) was implanted into the right CeLC—2.0 mm rostrally, 4.2 mm laterally, and 7 mm ventrally[11, 24, 40, 41] or the right ventrolateral periaqueductal grey(vlPAG) -7.6 mm rostrally, -0.5 mm laterally, and 5 mm ventrally [42–45] toward the bregma[46] and fixed to the skull with dental acrylic. A dummy cannula (RWD Life Science, Shenzhen, China) that inserts into the guide cannula, served to reduce the incidence of occlusion. Then animals were returned to their cages and housed individually to recover for one week prior to the next experiment.

For drug infusion, the various solutions were injected into the CeLC or the vlPAG through an injector (RWD Life Science, Shenzhen, China), which extended 0.5 mm beyond the guide cannula to target CeLC or vlPAG. The injector was attached to a 10 μl Hamilton syringe via polyethylene tubing (PE-10), and the solution was infused with a pump at 0.15 μl/min for 2 min. Waiting for another 2 min, the injection cannula was gently removed.

### Electrophysiology experiment

#### Slice preparation

Sprague Dawley male rats anaesthetized with sodium pentobarbital (50 mg/kg, i.p.) were decapitated, and coronal midbrain slices (350 μm) containing PAG tissue were prepared by a vibrating microtome (VT1000S, Leica Microsystems, Nussbloch, Germany) in cutting solution at 4°C. The dissection solution contained (in mM) 213 sucrose, 3 KCl, 1 NaH_2_PO_4_, 0.5 CaCl_2_, 5 MgCl_2_, 26 NaHCO_3_ and 10 glucose. The slices were incubated in the artificial cerebrospinal (ACSF) at 34°C for at least 1h. A single brain slice was then placed onto the recording chamber and perfused with 37°C running ACSF that was equilibrated with 95%O_2_ and 5%CO_2_ at a rate of 2 ml/min. The ACSF perfusion solution contained (in mM) 125 NaCl, 5 KCl, 1.2 NaH_2_PO_4_, 2.6 CaCl_2_, 1.3 MgCl_2_, 26 NaHCO_3_, and 10 glucose[47, 48]. Only 2–3 brain slices per animal were used, and only 1 neuron was recorded in each slice.

#### Whole-cell patch-clamp recording

A whole cell voltage-clamp technique was used to record mEPSCs in the vl-PAG neurons as previously described [11, 47–49]. Borosilicate glass capillaries (1.5 mm outer diameter, 1.0 mm inner diameter; WPI, USA) were pulled to make the recording pipettes (impedance was 4 MΩ- 6 MΩ) filled with the following internal solution (in mM):145 KCl, 5 NaCl, 10 HEPES, 5 EGTA,4 Mg-ATP, and 0.3 Na_3_-GTP, pH adjusted to 7.3 with KOH. A 5 min equilibration period was allowed to reach a steady state after whole cell access was established. Cells that exhibited any obvious (> 15%) change during the recording period were abandoned. A HEKA EPC-10 amplifier (HEKA, Lambrecht, Germany) and patchmaster software (Molecular Devices, Sunnyvale, Calif, USA) were used for data acquisition and analysis. mEPSCs were obtained at a holding potential of -70 mV (in the presence of 50 μM picrotoxin and 1 μM tetrodotoxin) and measured 10 min before and 15 min after drug application[50]. A 5min fixed length of traces was analyzed for frequency and amplitude changes using Mini Analysis program 6.0 (Synaptosoft Inc, Fort Lee, NJ, USA) and pCLAMP10 software (Molecular Devices, Sunnyvale, Calif, USA).

### Statistics

The sample size used in experiments was based on our previous studies [11]. Based on this calculation, we have chosen to increase the sample size to ensure accurate data. Data are presented as Mean ± SD. Using the D’Agostino and Pearson omnibus normality test, data were normal distribution and parametric statistics were applied. Homogeneity of variance was proved by the Bartlett's test. Western blot analysis was assessed using one-way analysis of variance (ANOVA) followed by Bonferroni *post-hoc* comparisons. Behavioral test in all experiments were analyzed by two-way repeated measures ANOVA (time and drug as variables) followed by Bonferroni multiple *post-hoc* comparisons. The effect of KN93 on the change of frequency and amplitude of mEPSCs in vl-PAG cells (added in the PAG slices) were analyzed by Student’s *t*-tests (paired), and in PAG cells (micro-infused into CeLC) were analyzed by one-way ANOVA followed by the Bonferroni *post hoc* test. All graphs and statistical analysis were performed using GraphPad Prism6.0 (GraphPad Software, San Diego, CA). Significant differences were defined as *P* < 0.05.

## Results

### Intra-CeLC KN93 micro-injection reversed fentanyl-induced hyperalgesia and inhibited the CaMKIIα activation in CeLC, vlPAG, RVM and spinal cord in OIH rats (Experiment 1)

**Fig 2:**
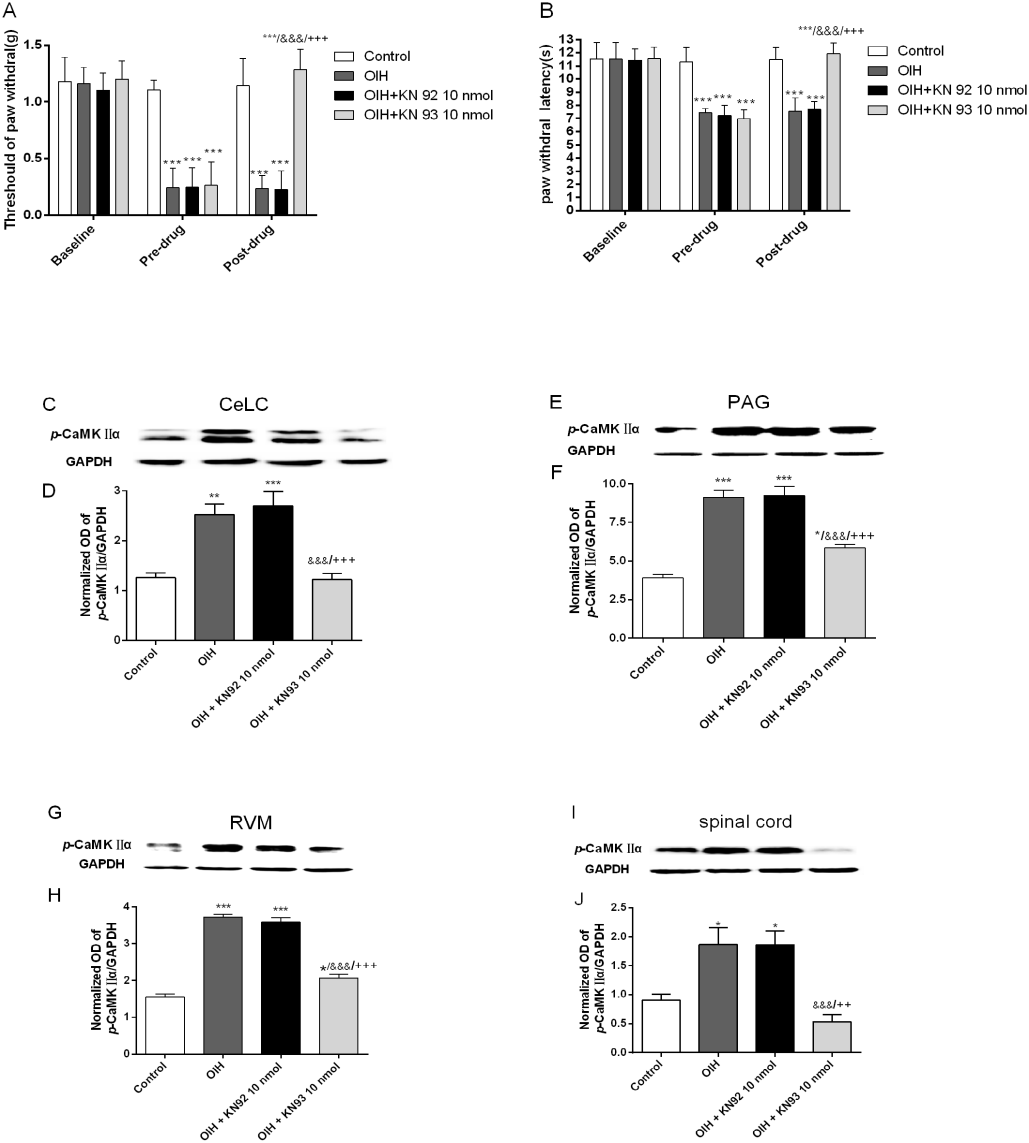
Intra-CeLC KN93 micro-injection reversed fentanyl-induced hyperalgesia and inhibited the CaMKIIα activation in CeLC, vlPAG, RVM and spinal cord in OIH rats. (A-B) Bar graphs show the measurements of the mechanical threshold of paw withdrawal (A) and thermal paw withdrawal latency (± SD) collected at baseline (before Cannulation), pre-drug (6h after the last injection of fentanyl or saline) and then post-drug (0.5 h after microinjection). (C-J) Bar graphs show the mean relative density of *p*-CaMKIIα to GAPDH in CeLC (C- D), vlPAG (E-F), RVM (G-H) and spinal cord (I-J). Control, OIH, OIH+KN92 45 nmol, and OIH+KN93 45 nmol group received 50% DMSO (vehicle), 50%DMSO (vehicle), KN92 10 nmol and KN93 10 nmol respectively through the CeLC cannula. *, compared with Control group; &, compared with OIH group; +, compared with OIH+KN92 45 nmol group, n = 6 for each group; One symbol: *p* < 0.05, Two symbols: *p* < 0.01, Three symbols: *p* < 0.001.

To investigate the effect of intra-CeLC KN93 (CaMKIIα antagonist) micro-injection on the CaMKIIα activity in the proposed ‘CeLC-PAG-RVM-spinal cord’ pathway and the behavioral hyperalgesia induced by fentanyl, rats were first implanted with CeLC cannulas before the induction of OIH. Then the Control group (n = 6) and OIH group (n = 6) received 50% DMSO (vehicle) 0.3 μl. OIH+KN92 10 nmol group (n = 6) and OIH+KN93 10 nmol group (n = 6) received equal volume (0.3 μl) of KN92 10 nmol and KN93 10 nmol respectively 6.5 h after the last injection of fentanyl. Confirming previous reports [11], our data revealed that the established fentanyl-induced hyperalgesia was rapidly attenuated by KN93, while the same treatment with KN92 (kinase-inactive chemical analogue) or vehicle did not alter the pain threshold (F = 34.63, *p* < 0.001, two-way repeated measures ANOVA followed by Bonferroni multiple *post-hoc* comparisons; Fig 2A and 2B). Correlated with the behavioral effects, intra-CeLC KN93(10 nmol) application not only attenuated the increased *p-*CaMKIIα expression in the CeLC area, but also the PAG, RVM and the spinal cord regions induced by fentanyl. While KN92 or vehicle had little effect (one-way ANOVA followed by Bonferroni *post-hoc* comparisons; Fig 2C–2J). It implied that CeLC should be the upstream of the PAG, RVM and spinal cord in modulating fentanyl-induced hyperalgesia.

### Intrathecal KN93 injection attenuated fentanyl-induced hyperalgesia and decreased the level of *p*-CaMKIIα in spinal cord but not CeLC in OIH rats (Experiment 2)

**Fig 3:**
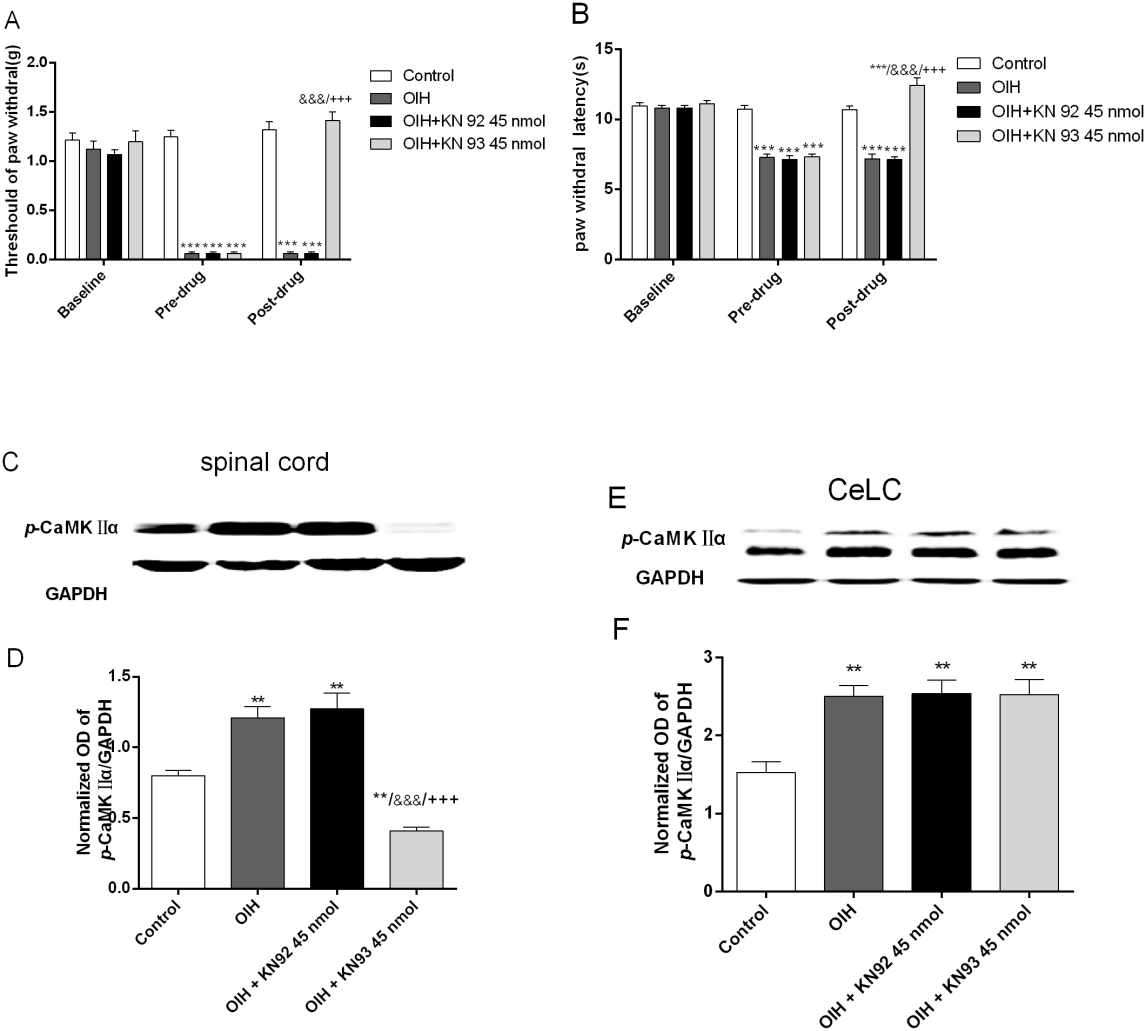
Intrathecal KN93 injection attenuated fentanyl-induced hyperalgesia and decreased the level of *p*-CaMKIIα in spinal cord but not CeLC in OIH rats. (A, B) Graphical display of the mechanical threshold of paw withdrawal (A) and thermal paw withdrawal latency (B) collected at baseline, pre-drug (6 h after the last injection of fentanyl or saline), and post-drug (0.5 h after Intrathecal injection). (C-F) Representative Immunoblots of activated CaMKII*α* (*p*-CaMKII*α*) and histogram of relative density of *p*-CaMKII*α* to GAPDH in the spinal cord (C-D) and CeLC (E-F). Control, OIH, OIH+KN92 45 nmol, and OIH+KN93 45 nmol group received i.t. injection with 50% DMSO (vehicle), 50% DMSO (vehicle), KN92 10 nmol and KN93 10 nmol respectively. Results are expressed as mean ± SD; *, compared with Control group; &, compared with OIH group; +, compared with OIH+KN92 45 nmol group, n = 6 for each group; One symbol: *p* < 0.05, Two symbols: *p* < 0.01, Three symbols: *p* < 0.001.

According to our former results that CeLC might be the upstream of the PAG, RVM and spinal cord in modulating OIH, the next experiment was performed to test whether spinal cord has a reverse effect on amygdala CaMKIIα signal. In line with the previous studies [12, 51], inhibition of spinal CaMKIIα by an i.t. injection of KN93 (45 nmol) attenuated behavioral hyperalgesia induced by fentanyl (n = 6 for each group, *p* < 0.001, two-way repeated measures ANOVA followed by Bonferroni multiple *post-hoc* comparisons; Fig 3A and 3B). While KN92 and vehicle both had little effect on nociceptive behaviors. After behavioral test, the spinal cord and CeLC tissue is dissected to perform Western blot analysis to explore whether i.t. KN93 could reverse the CaMKIIα activation in CeLC in OIH rats. Results shown that inhibition of spinal CaMKIIα by KN93 (45 nmol) could not attenuate the increased level of CeLC *p*-CaMKIIα in OIH rats (one-way ANOVA followed by Bonferroni *post-hoc* comparisons; Fig 3C–3F), demonstrating that amygdala can facilitate downstream spinal CaMKIIα activation during the maintenance of OIH, on the contrary, the spinal cord exhibit little reverse activation on amygdala CaMKIIa signal.

### Intra-vlPAG KN93 micro-injection reversed fentanyl-induced hyperalgesia and inhibited CaMKIIα activation in PAG, RVM and spinal cord but not in CeLC (Experiment 3)

**Fig 4:**
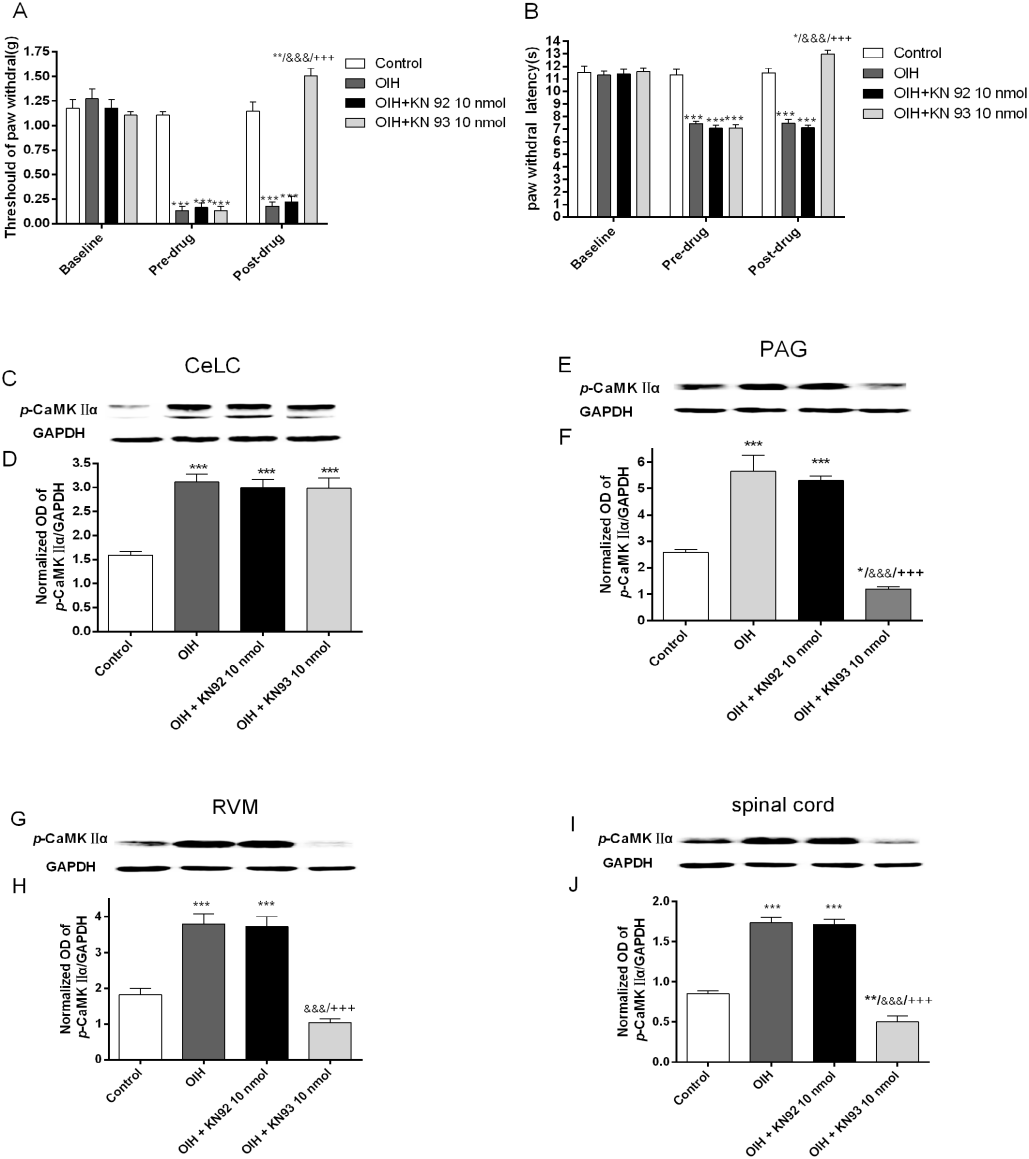
Intra-vlPAG KN93 micro-injection reversed fentanyl-induced hyperalgesia and inhibited CaMKIIα activation in PAG, RVM and spinal cord but not in CeLC. (A-B) Graphical display of the mechanical threshold of paw withdrawal (A) and thermal paw withdrawal latency collected at baseline (before Cannulation), pre-drug (6 h after the last injection of fentanyl or saline) and then post-drug (0.5 h after microinjection). (C-J) Representative immunoblots of activated CaMKII*α* (*p*-CaMKII*α*) and histogram of relative density of *p*-CaMKII*α* to GAPDH in the CeLC (C-D), vlPAG (E-F), RVM (G-H) and spinal cord (I-J). Control group (n = 6) and OIH group (n = 6) received 50% DMSO (vehicle) 0.3 μl. OIH+KN92 10 nmol group (n = 6) and OIH+KN93 10 nmol group (n = 6) received KN92 10 nmol and KN93 10 nmol dissolved in 0.3 μl of 50% DMSO respectively through an intra-vlPAG cannula 6.5 h after the last injection of fentanyl. Results are expressed as mean ± SD; *, compared with Control group; &, compared with OIH group; +, compared with OIH+KN92 45 nmol group, n = 6 for each group; One symbol: *p* < 0.05, Two symbols: *p* < 0.01, Three symbols: *p* < 0.001.

Since amygdala can facilitate downstream spinal CaMKIIα activation during the maintenance of OIH, we then explored if this effect was exerted via a ‘CeLC-PAG-RVM-spinal cord’ descending pathway. To confirm whether *p-*CaMKIIα in vlPAG is involved in fentanyl-induced hyperalgesia and whether there is connection between PAG and other regions including CeLC, RVM and spinal cord in OIH, intra-vlPAG injection of KN93 was performed in OIH rats. Control group (n = 6) and OIH group (n = 6) received 50% DMSO (vehicle) 0.3 μl. OIH+KN92 10 nmol group (n = 6) and OIH+KN93 10 nmol group (n = 6) received KN92 10 nmol and KN93 10 nmol [52, 53] respectively through an intra-vlPAG cannula 6.5 h after the last injection of fentanyl. As shown in Fig 4, the established behavioral hyperalgesia induced by fentanyl was rapidly reversed by Intra-vlPAG injection of KNN93, while KN92 and vehicle did not alter the pain threshold (*p* < 0.001, two-way repeated measures ANOVA followed by Bonferroni multiple *post-hoc* comparisons; Fig 4A and 4B). Correlated with the behavioral changes, intra-vlPAG KN93 (not KN92 and vehicle) injection decreased *p-*CaMKIIα expression in the PAG, RVM and the spinal cord but not in CeLC compared with control (one-way ANOVA followed by Bonferroni *post-hoc* comparisons; Fig 4C–4J). These results proved that PAG might be the upstream of RVM and spinal cord and the downstream of CeLC in modulating fentanyl-induced hyperalgesia.

### Reversion of increased mEPSCs in vlPAG neurons from the OIH rats by KN93 (added in the ACSF) (Experiment 4)

**Fig 5:**
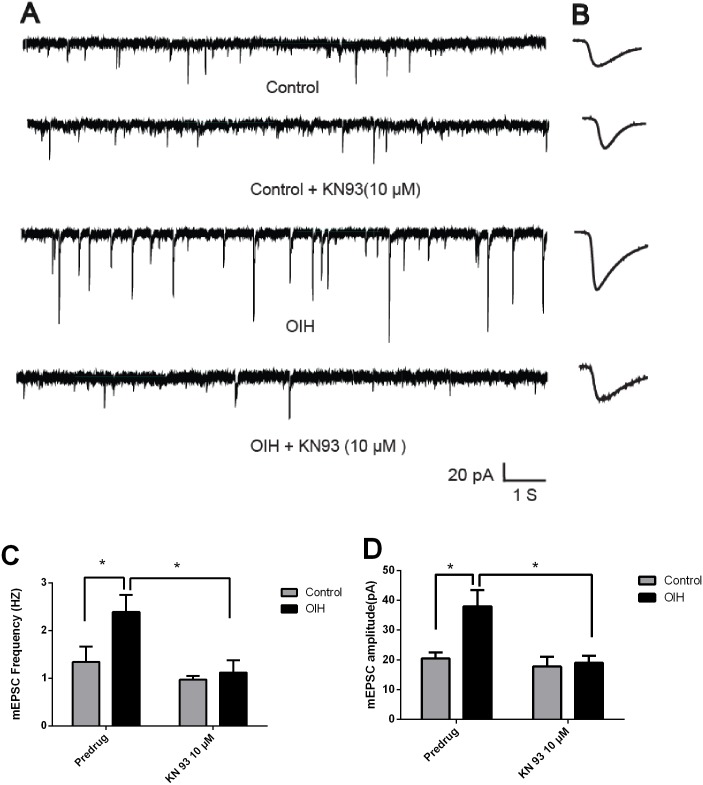
KN93 (added in the ACSF) reversed the enhanced mEPSCs in vlPAG neurons from the OIH rats. (A) mEPSCs recordings in control and OIH group at baseline and after the application of KN93. (B) Individual traces (average) of mEPSCs obtained from respective recordings. Calibration: 1 s, 20 pA. (C-D). Bar graphs showing the frequency (C) and amplitude (D) of mEPSCs in vlPAG neurons in control and OIH group (12 h post induction). Results are expressed as mean ± SD; n = 6 for each group; *, *p* < 0.05.

The analysis of mEPSCs is a well-established electrophysiological approach to determine pre- versus post-synaptic mechanisms. Presynaptic changes at the transmitter release site affect mEPSCs frequency, whereas changes at the postsynaptic membrane alter mEPSCs amplitude (quantal size) [41, 54]. Previous studies from our laboratory have shown that KN93 significantly decreased both the frequency and amplitude of mEPSCs in CeLC neurons from the OIH rats, suggesting that CaMKIIα regulates synaptic transmission in CeLC neurons through pre- and postsynaptic mechanisms [11]. In addition, anatomical studies have demonstrated direct reciprocal neurons projections between the amygdala and the PAG [30, 31]. Therefore, mEPSCs were recorded in the PAG neurons to determine whether there were differences between the control rats and OIH rats in the synaptic transmission, and whether CaMKIIα modulates synaptic transmission in PAG neurons in OIH rats. Results shown that KN93 (10 μM, added in the ACSF) [55–57] significantly reversed both the increased frequency (Fig 5C) and amplitude (Fig 5D) of mEPSCs recorded from the vlPAG neurons in slices from OIH rats (12 h post OIH induction), but not those from the control rats (paired *t*-tests; n = 6).

### Inhibition of mEPSCs in vlPAG neurons from the OIH rats by intro-CeLC injection of KN93 (Experiment 5)

**Fig 6:**
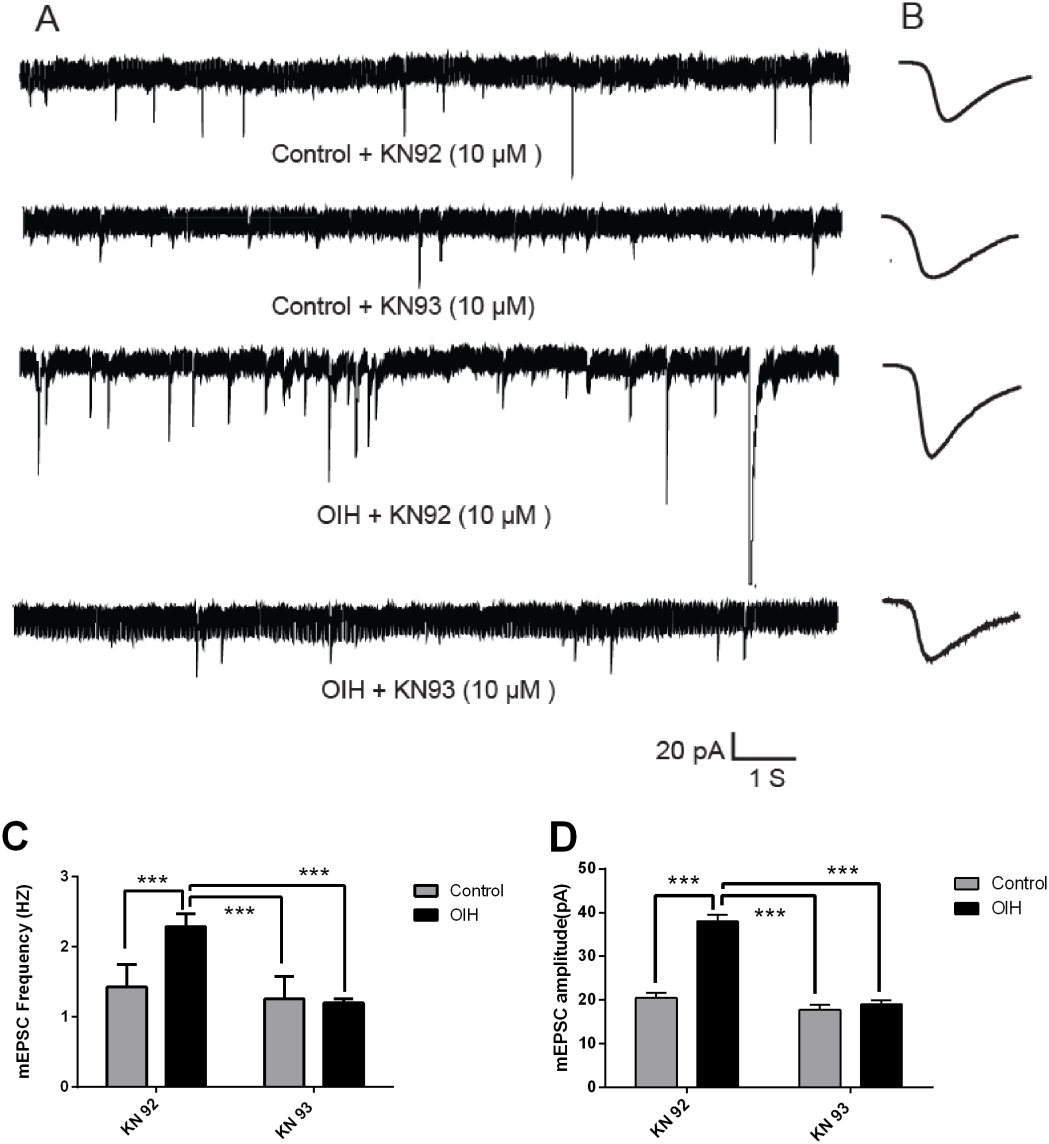
Inhibition of mEPSCs in vlPAG neurons from the OIH rats by intro-CeLC injection of KN93. (A) mEPSCs recordings in control and OIH group after the application of KN92 or KN93. (B) Individual traces (average) of mEPSCs obtained from respective recordings. Calibration: 1 s, 20 pA. (C-D) Bar graphs showing the frequency (C) and amplitude (D) of mEPSCs from vlPAG neurons in slices from control and OIH group (12 h post induction). Results are expressed as mean ± SD; n = 8–11 for each group; *, *p* < 0.05, **, *p* < 0.01.

To further confirm whether in vivo inhibition of CeLC CaMKIIα activity has a direct effect on the enhanced synaptic transmission of vlPAG neurons in OIH rats, mEPSCs was recorded in vlPAG neurons after intro-CeLC injection of KN93(10 nmol)[11]. It was shown that, compared with control (non-OIH rats), intro-CeLC injection of KN93, but not KN92, significantly decreased the frequency (Fig 6C) and amplitude (Fig 6D) of mEPSCs recorded from the vlPAG neurons in slices from OIH rats (12 h post OIH induction) (one-way ANOVA followed by Bonferroni *post-hoc* comparisons; n = 8–11).

## Discussion

To our knowledge, this is the first report addressing the role of CaMKIIα in the CeLC, PAG, RVM and spinal cord as a whole in the modulation of fentanyl-induced hyperalgesia. Our data reveal that, CaMKIIα may modulate OIH via a CeLC-PAG-RVM-spinal cord descending facilitative pain pathway. These findings may provide novel insight into the mechanisms underlying fentanyl-induced hyperalgesia.

In this study, results shown that inhibition of CaMKIIα activity at any level of the ‘CeLC-PAG-RVM-spinal cord’ pathway can reverse the behavioral hyperalgesia induced by fentanyl. However, only intervention at upstream of this pathway can modulate the downstream molecule (CaMKIIα) activation and synaptic potentiation, but not vice versa. A possible explanation of this phenomenon is that there is a pronociceptive facilitative cascade from CeLC to PAG, RVM, and spinal cord. And CaMKIIα is the key molecule for this facilitative cascade.

PAG, one of the core components of the descending pain-modulatory system, has been proved to project to the rostral ventromedial medulla (RVM) and the spinal cord. The PAG also receives inputs from the CeLC, which is involved in mediating top-down analgesia[58–60]. It is now believed that the CeLC produces descending modulation of pain through peptidergic projections to the PAG and RVM [28, 58, 61]. Reports have shown that the protein kinase C-δ- positive neurons and somatostatin-expressing neurons are the major population of the lateral subdivision of the central amygdala(CeLC) neurons[31, 62]. Whether CeLC neurons that project to vlPAG have the same cell types will be further studied. The modulatory facilitatory or inhibitory effect of PAG on pain is generated concomitantly with the changes of the RVM neurons that related the facilitative or inhibitory modulation of pain[63, 64]. The RVM can both facilitate and inhibit pain through recruitment of RVM on-or off-cells, respectively [65–69]. The pronociceptive on-cells and antinociceptive off-cells in RVM region that project to spinal cord offer a neuronal condition for facilitation and inhibition pain regulation from the descending pain pathway[66]. It is reported that pain threshold might change with the balance between the on-cells and off-cells populations in RVM[65] and sustained exposure to morphine resulted in an increased proportion of on-cells, which might contribute to morphine-induced paradoxical pain[70]. So we speculate that synaptic facilitation of PAG manifested by the enhancement of mEPSCs, likely acting through the RVM on-cells activity, contributes to the OIH modulation derived from CeLC, driving the exaggerated behavioral responses and increased spinal neuronal excitability to non-noxious stimulations observed in OIH rats. Of course, the activity and the proportion of on- or off-cells recorded from the RVM under OIH condition need to be identified in future studies.

mEPSCs, a well-established electrophysiological approach to determine pre- or postsynaptic mechanisms, is assumed to represent the spontaneous release of individual vesicles or quanta of neurotransmitter from the presynaptic membrane[71]. Changes at the postsynaptic membrane are known to alter quantal size(mEPSCs amplitude), whereas presynaptic changes at the transmitter release site affect the frequency of mEPSCs[72]. Consistent with previous report that both pre- and postsynaptic CaMKIIα are necessary for the induction of synaptic plasticity[73], our results shown that CaMKIIα inhibitor added in ACSF or injected into CeLC significantly reversed or inhibited both the increased frequency and amplitude of mEPSCs recorded on vlPAG neurons in slices from OIH rats. So, we can safely speculate that CaMKIIα modulated the synaptic plasticity in vlPAG neurons involved both pre- and postsynaptic mechanisms.

CaMKIIα is expressed at presynaptic nerve terminal as well as the postsynaptic density [74]. At presynaptic location, transient receptor potential vanilloid type 1 channel (TRPV1) has been proved plays an important role in OIH [75–78] and it can interact with CaMKIIα physiologically and pharmacologically [79, 80], thus forming possible feed-forward loops[81] to enhances vesicle motility and facilitates presynaptic spontaneous neurotransmitter release [74]. This mechanism may underlie the increased frequency observed in vlPAG neurons. At postsynaptic location, N-methyl-D-aspartatereceptor (NMDAR), which plays an important role in the development of OIH [10, 82], can also interact with CaMKIIα to form a positive feed-forward to facilitate synaptic potentiation. This mechanism may contribute to the amplitude increase of the mEPSC recorded in the present study. Actually, it has reported that opioids promote the activation of CaMKIIα in PAG synaptosomes probably via activation of NMDA receptors [83–86].

It is shown that only right amygdala develops pain-related plasticity that is coupled to pain facilitation in the arthritis pain model [87]. And only ventral lateral part of PAG (vlPAG) has been proved functionally connected to pain circuit in the RVM [43, 88]. In addition, there is a strong contralateral projection of pain pathway [22], so behavioral tests in all the experiments in the present study were performed in the left plantar of animal, while analyzing the level of phosphorylation of CaMKIIα (*p-*CaMKIIα) and recording synaptic transmission in the right CeLC and vlPAG.

The dose of fentanyl used in pediatric patients is usually about (2–3) μg/kg during surgery, and in this experiment the dosage of fentanyl (60 μg/kg, 4 times, 15 min intervals, s.c.) was based on our previous study [11]. According to equivalent dose conversion between the species, this dose (240μg/kg) was used to mimic the high dose used in pediatric patient’s surgeries. In line with the observation that up to postnatal day 21 in rats, the RVM solely has the facilitatory effect on spinal pain transmission but that after this age (postnatal day 28 to adult), the facilitatory effect of the RVM turns to biphasic facilitation and inhibition [89]. Moreover, most relevant studies have only focused on the mechanisms of OIH in adult rats, but we cannot deny the increased morbidity rate and opioid exposure in the adolescents. Therefore, we used adolescent rats (about 4 weeks) in all the experiments. In this study, all the experiments are conducted on rats without obvious peripheral nerve injury or inflammation. Nevertheless, in clinics, opioids are mostly administered to patients suffering from chronic or acute pain disorders, further studies of an animal model of surgical pain are needed to test.

Although the mechanisms by which CaMKIIα regulates OIH are unknown, we suppose CaMKIIα may modulate OIH via a CeLC-PAG-RVM-spinal cord descending pain pathway. However, In addition to the PAG-RVM system, the dorsal reticular nucleus (DRt) and caudal lateral ventrolateral medulla (VLM) have also been reported to be involved in modulation of descending pain[65]. Neurons that project along the prefrontal cortex (PFC)–amygdala–PAG pathway have been implicated in mediating descending pain[46] and the PAG also receives inputs from the hypothalamus, PFC, and anterior cingulate cortex (ACC), which plays a major role in mediating pain[60]. Thus, we cannot deny the role of hypothalamus, PFC, ACC, DRt and VLM on this descending pathway. In a word, we hope that our study provides novel insight into the mechanisms underlying fentanyl-induced hyperalgesia.
